# Remote Ischemic Preconditioning (RIPC) Modifies the Plasma Proteome in Children Undergoing Repair of Tetralogy of Fallot: A Randomized Controlled Trial

**DOI:** 10.1371/journal.pone.0122778

**Published:** 2015-03-31

**Authors:** Michele Hepponstall, Vera Ignjatovic, Steve Binos, Chantal Attard, Vasiliki Karlaftis, Yves d’Udekem, Paul Monagle, Igor E. Konstantinov

**Affiliations:** 1 Murdoch Childrens Research Institute, Melbourne, Australia; 2 Cardiac Surgery Unit and Cardiology, Royal Children’s Hospital, Melbourne, Australia; 3 Department of Paediatrics, The University of Melbourne, Melbourne, Australia; 4 Department of Environment and Primary Industries, Bioscience Research Division, Melbourne, Australia; TNO, NETHERLANDS

## Abstract

**Background:**

Remote ischemic preconditioning (RIPC) has been applied in paediatric cardiac surgery. We have demonstrated that RIPC induces a proteomic response in plasma of healthy volunteers. We tested the hypothesis that RIPC modifies the proteomic response in children undergoing Tetralogy of Fallot (TOF) repair.

**Methods and Results:**

Children (n=40) were randomized to RIPC and control groups. Blood was sampled at baseline, after cardiopulmonary bypass (CPB) and 6, 12 and 24h post-CPB. Plasma was analysed by liquid chromatography mass spectrometry (LC-MS) in an untargeted approach. Peptides demonstrating differential expression (p<0.01) were subjected to tandem LC-MS/MS and protein identification. Corresponding proteins were identified using the NCBI protein database. There was no difference in age (7.3±3.5vs6.8±3.6 months)(p=0.89), weight (7.7±1.8vs7.5±1.9 kg)(p=0.71), CPB time (104±7vs94±7 min)(p=0.98) or aortic cross-clamp time (83±22vs75±20 min)(p=0.36). No peptides were differentially expressed at baseline or immediately after CPB. There were 48 peptides with higher expression in the RIPC group 6h post-CPB. This was no longer evident at 12 or 24h, with one peptide down-regulated in the RIPC group. The proteins identified were: inter-alpha globulin inhibitor (42.0±11.8 vs 820.8±181.1, p=0.006), fibrinogen preproprotein (59.3±11.2 vs 1192.6±278.3, p=0.007), complement-C3 precursor (391.2±160.9 vs 5385.1±689.4, p=0.0005), complement C4B (151.5±17.8 vs 4587.8±799.2, p=0.003), apolipoprotein B100 (53.4±8.3 vs 1364.5±278.2, p=0.005) and urinary proteinase inhibitor (358.6±74.9 vs 5758.1±1343.1, p=0.009). These proteins are involved in metabolism, haemostasis, immunity and inflammation.

**Conclusions:**

We provided the first comprehensive analysis of RIPC-induced proteomic changes in children undergoing surgery. The proteomic changes peak 6h post-CPB and return to baseline within 24h of surgery.

**Trial Registration:**

ACTR.org.au ACTRN12610000496011

## Introduction

First described in 1986 by Murray et al [1], ischemic preconditioning (IPC) is a phenomenon whereby brief periods of ischemia can provide protection against subsequent prolonged ischemia. The concept has since evolved into a clinically applicable protection known as remote IPC (RIPC) where ischemia of peripheral tissue, including skeletal muscle, could protect distant organs from ischemia-reperfusion (IR) injury [2]. This can be applied non-invasively in various clinical scenarios.

The first clinical application of RIPC in humans was reported in a randomized clinical trial (RCT) in children undergoing heart surgery and demonstrated cardiopulmonary protection with decreased inotrope requirement, decreased airway resistance and decreased troponin levels [3]. Subsequently, the RIPC-induced myocardial protection was demonstrated in other RCTs [4–8]. Recent meta-analysis of the RCTs also demonstrated significant myocardial protection by the RIPC against IR injury in patients undergoing heart surgery with cardioplegic arrest and cardiopulmonary bypass (CPB) [9].

In contrast to these studies with positive findings, the impact of RIPC on myocardial protection in the setting of chronic hypoxia in children is less clear. Our two recent RCTs demonstrate that in chronically hypoxic children with cyanotic heart disease, a high proportion of proteins are in phosphorylated form and RIPC does not further enhance phosphorylated protein signalling in myocardium or circulating leukocytes in children undergoing Tetralogy of Fallot (ToF) repair [10] or provide myocardial protection [11]. To dissect the molecular impact of the RIPC in children with cyanotic heart disease, we performed a further proteomic analysis of the RIPC-induced changes in plasma of children undergoing repair of ToF [10].

## Methods

The protocol for this trial and supporting CONSORT checklist are available as supporting information; see S1 CONSORT Checklist and S1 Protocol.

This double blind RCT was approved by the Royal Children’s Hospital Human Research Ethics committee and conducted in accordance with the National Health and Medical Research Council of Australia Statement on ethical conduct in human research. Informed, written consent was obtained from the parents of the patients involved in the study. The RCT was registered with the Australian and New Zealand Clinical Trials Registry. Registry number ACTRN12610000496011.

### Patients

**Fig 1:**
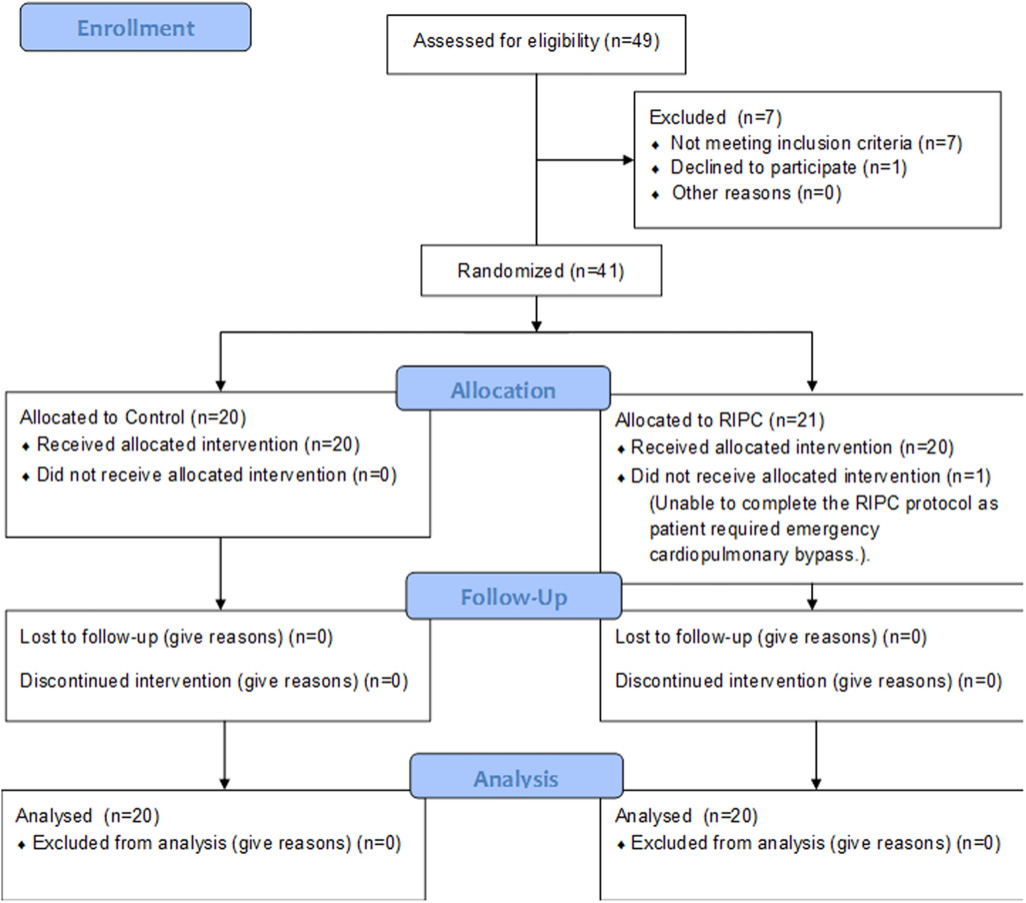
CONSORT diagram describing flow of the participants though the enrolment, allocation, follow up and analysis phases of the trial.

All patients who underwent elective repair of ToF from July 2010 to June 2012 were eligible to participate in the study if they were having their first surgery for complete ToF repair (Fig. 1). Eligible patients were enrolled by the surgeons.

Patients with chromosomal abnormalities, congenital lung malformations and haematological disorders were excluded. Patients (n = 40) aged between 1.8 months and 14 months (Table 1) were randomized into control group (n = 20) and RIPC group (n = 20).

**Table 1:** Demographic data for the Control and RIPC groups.

	Control	RIPC	p value
**Patients**	20	20	
**Gender**	9M, 11F	11M, 9F	0.75
**Weight (kg)**	7.7 ± 1.8 (5.0–11.6)	7.5 ± 1.9 (5.1–10.8)	0.71
**Age (months)**	7.3 ± 3.5 (3.3–14.2)	6.8 ± 3.6 (1.8–15.1)	0.89
**Cardiopulmonary bypass time (CPB)(mins)**	104 ± 7 (80–158)	94 ± 7 (55–186)	0.98
**Total aortic cross-clamp time (mins)**	83 ± 22 (56–131)	75 ± 20 (43–108)	0.36
**Preoperative oxygen saturations (%)**	92.6 ± 5.6 (80–98)	87.3 ± 9.1 (70–100)	0.04
**Total CPB prime volume (mL)**	472 ± 12 (400–542)	461 ± 12 (356–556)	0.56

Sample size was determined based on our successful study of RIPC in children which was amply powered to detect differences in clinical and molecular measures [3]. Injury was deemed to be diminished by RIPC on the basis of reduced troponin I release, thus for a power of 0.8, alpha of 0.05 and a sigma of 3 the sample size required is calculated as n = 16. Thus a sample size of n = 20 per group would yield ample statistical power.

### Randomisation

Blocked randomisation (block size = 10) was used to allocate patients. The randomisation was generated by a member of the laboratory research staff using SPSS statistical software and concealed in sequentially numbered sealed envelopes. The envelopes were opened in numerical order immediately prior to the RIPC or sham intervention. The research co-ordinator assigned the interventions to patients.

### Remote ischemic preconditioning protocol

The RIPC protocol consisted of 4 cycles of 5 minutes ischaemia, followed by 5 minutes of reperfusion applied to the patient’s leg in the operating theatre after induction of anaesthesia prior to sternotomy [3]. A standard blood pressure cuff (Welch Allen, Germany) was applied to the thigh and inflated to a pressure exceeding systolic pressure by 20 mmHg, determined by blood pressure monitored via an arterial line. This was followed by deflation of the cuff. Interruption and restoration of blood flow was confirmed by pulse oximetry applied distal to the blood pressure cuff. Control patients had sham placement of the blood pressure cuff on a lower limb without inflation. All clinical staff and patients’ families were blinded to the allocation.

### Cardiac surgery

Cardiac surgery via median sternotomy was performed using standard anaesthesia, cardioplegic arrest and CPB as described previously [10].

### Blood sampling

**Fig 2:**
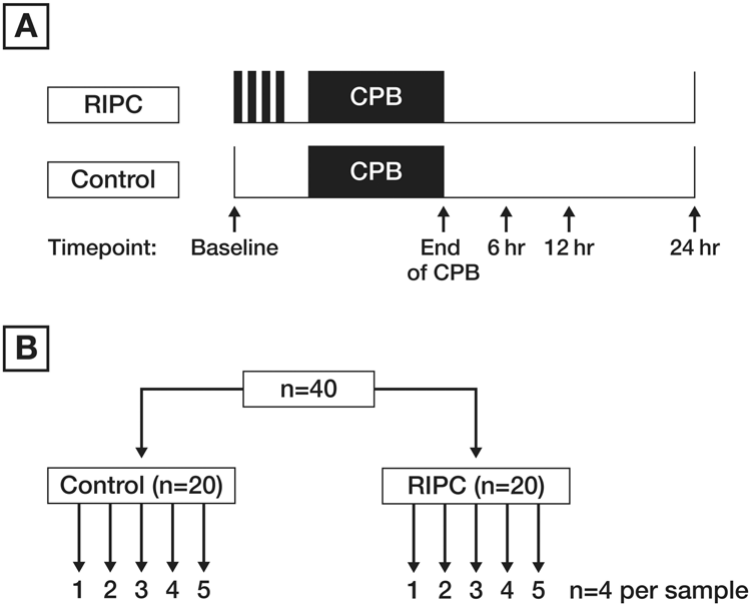
Time points of the sample collection in panel A. Pooling of the plasma samples at each time point in panel **B.** Plasma from all 20 patients of one group was pooled into 5 samples (each representing plasma of 4 patients).

A total of 5 blood samples were collected from each patient. Blood samples were collected prior to RIPC or sham, at the cessation of CPB and at six, 12 and 24 hours after CPB (Fig. 2A). Blood (1.4 mL) was collected in S-Monovette tubes (Sarstedt, Australia), containing one volume per nine volumes of blood of 0.106mol/L (3.2%) trisodium citrate anticoagulant. Plasma was obtained by centrifugation at 10°C for 10 min at 3000 rpm (Megafuge 1.0R, Heraeus) and stored at -80°C for batch testing.

### Preparation of the plasma samples

For each of 5 time points, 40 samples (Contol, n = 20 and RIPC, n = 20) were collected and the samples from each group were pooled into 5 samples, so that each of those pooled 5 samples was representative of 4 patients in that group (Fig. 2B). Prior to pooling, 100 uL of each individual sample was transferred into tubes containing 4 uL protease cocktail inhibitors (Sigma Aldrich) to stop degradation of the proteins by endogenous proteases. Samples were filtered through 45μm spin filters.

Ion exchange chromatography was employed to exclude albumin and IgG from the plasma based on the isoelectric point of these proteins. Plasma samples were passed through a strong cation exchange column (50 mM Tris HCl pH 6.8) as well as a strong anion exchange column (50 mM sodium acetate at pH 5.2) (Pierce, Thermo Scientific) according to the manufacturer’s instructions. The proteins from the strong cation exchange columns and the strong anion exchange columns were combined for analysis.

### Quantification of protein content

The Bradford assay [12] (Bio-Rad, Hercules, CA, USA) was used to determine the protein content of each sample by comparing the absorbance at 595 nm against a standard curve of Bovine serum albumin dissolved in 50 mM Tris HCl, 50 mM sodium acetate 0.75 M NaCl, the same buffer that the samples were in.

### Trichloroacetic acid (TCA) precipitation

Protein precipitation was carried out to concentrate the sample and dissolve the protein in a buffer suitable for trypsin digestion. The precipitation involved adding one volume of 100% (w/v) TCA to four volumes of protein sample.

The samples were incubated at 4°C for 10 minutes and pelleted by centrifuged at 14 000 g for five minutes. The supernatant was removed and the pellet washed with 200 μL cold acetone and centrifuged at 14 000 g for five minutes. The acetone wash was repeated. Pellets were dried by decanting off the acetone and evaporating the remaining acetone. The protein pellets were redissolved in 60 μL of 6 M Urea.

### In-solution trypsin digestion

Trypsin digestion was performed on fifty micrograms of protein from each sample [13].

### Sample preparation for mass spectrometry

Peptides were cleaned and concentrated by Solid Phase Extraction. Samples were passed through 1 mL (10 mg capacity) Oasis HLB extraction cartridges (Waters, Ireland). The columns were preconditioned with methanol and equilibrated with 3 mL 2% acetonitrile and 0.1% Trifluroacetic acid (TFA). The sample was equilibrated 1/50 with 100% acetonitrile and 1/100 TFA then loaded onto the column. The column was washed with 3 mL 2% acetonitrile and 0.1% TFA. Bound peptides were eluted with 1 mL 80% acetonitrile and 0.1% TFA. Samples were frozen in liquid nitrogen and lyophilised by freeze drying overnight. Lyophilised peptides were then resuspended in 200 uL 0.1% formic acid and sonicated for five minutes before being passed through 30 kD spin filters (Amicon Ultra 0.5 mL 30 kD) previously preconditioned with 0.1% formic acid and centrifuged at 14 000 rpm for 30 minutes. Sixty microliters of this solution was pipetted into glass vials in preparation for liquid chromatography, mass spectrometry (LC MS/MS).

### Mass spectrometry

LC MS/MS was carried out on an LTQ Orbitrap Velos (Thermo Scientific, West Palm Beach, FL, USA) equipped with a nanoelectrospray interface coupled to an Ultimate 3000 RSLC nanosystem (Dionex, Sunnyvale, CA, USA) using methodology we have previously described [13], with the following modifications. The 20 most intense peptide ions, with charge state ≥2 were isolated at a target value of 5000 for CID fragmentation. Dynamic exclusion was set at two repeat counts over 30 seconds and exclusion duration of 70 seconds. Raw data were collected using Thermo Xcalibur software (Thermo Scientific) and processed with Expressionist Refiner MS software (Genedata, Basel, Switzerland).

### Data analysis

Initial data processing was carried out using Expressionist Refiner MS (Genedata, Basel Switzerland) to align MS data, carry out noise reduction and for peak extraction (clustering). The peak area intensity measurements of precursor ions were analysed for statistical relevance using Genedata Analyst (AG, Basel, Switzerland).

At each time point, the control group was compared against the RIPC group. An effect size analysis was conducted to compare samples based on the calculation of fold change ratios. The analysis was set to identify those peptides that demonstrated a fold change of greater than or equal to two.

An unpaired t-test was conducted to identify those peptides that were significantly different between the two groups. Bootstrapping was included to determine the accuracy of sample estimates. In addition, a permutation Q-test was conducted to indicate a permutation Q-value which calculates a false discovery rate. Only p-values less than the false discovery were accepted as significant.

The results from the effect size analysis and t test analysis for each time point were combined in the form of Venn diagrams. Those peptides intersecting and therefore demonstrating fulfillment of both criteria, that is a fold change and statistically significant change were included in a second pass targeted MSMS analysis and subsequent data mining and protein identification. In addition, a two way ANOVA was performed to assess differences between groups across time.

### Targeted mass spectrometry

Three separate LC-MS/MS methods were implemented in an attempt to increase the chance of positive protein identification. For the targeted LC-MS/MS, the mass spectrometer was operated in data-dependent mode as described above using CID-LTQ but also in data-dependent mode using accurate mass, collision induced dissociation- Fourier transform (CID-FT) and higher-energy collisional dissociation- Fourier transform (HCD-FT) for MS/MS fragmentation at a resolution of 7500 in the Orbitrap. Independent of the MS/MS mode, the full MS scan was set at 60 000 resolution and the ten most intense peptide ions with charge states greater or equal to two were isolated at a minimum threshold value of 5000 from the assigned target list. A dynamic exclusion of two repeats over 30 seconds with an exclusion duration of 45 seconds was set.

### Data mining

Data from Thermo Xcalibur (Thermo Scientific) were imported into Proteome Discoverer version 1.3 (Thermo Fisher Scientific, West Palm Beach, FL, USA,) and Mascot v.2.3.01 (Matrix Science, London, UK). Data were imported with the following settings. Retention time limits between eight and 60 minutes. Lowest ion charge state was set as 2 and highest charge state at 6 for those ions most likely to represent peptides.

A MASCOT search was carried out on the latest version of the NCBInr human databases (National Centre for Biotechnology Information, Bethesda US) [13]. The identity of the target peptides was confirmed using the fragment information from the tandem MS in combination with matching retention time and m/z using the high resolution/accurate mass capabilities of the Orbitrap mass spectrometer. The characteristics of the peptides in the target lists, namely m/z, retention time, charge, and mass were compared against those from the Proteome Discoverer searches to identify matches within the databases. The fragmentation patterns from Proteome Discoverer and Genedata Analyst were compared to validate that the protein identifications were legitimate. Data were presented according to the CONSORT guidelines for reporting a RCT and the Paris guidelines for the presentation of proteomic data [14,15].

Validation of Complement C3 was conducted on the individual samples at all the time points using the Complement C3 human enzyme-linked immunosorbent assay (ELISA) kit (Abcam) According to the manufacturer’s directions. A Student’s t-test was performed at each time point to identify statistical differences between the groups with p<0.05 considered statistically significant.

## Results

Participant flow, through the enrolment, allocation and analysis phases of the trial is detailed in Fig. 1. There were no reported adverse events resulting from the RIPC or blood sampling protocols. There was no mortality.

Demographic data are presented in Table 1. With the exception of slightly lower preoperative saturations in the RIPC group, there were no differences in the baseline demographics between the two groups in terms of gender, weight, age, bypass time, total aortic cross clamp time or bypass circuit prime volume.

There were 335 964 clusters resulting from the MS analysis, of which 96 725 were selected for analysis based on a positive charge state ≥ 2. This criterion was chosen to select those clusters most likely to represent peptides.

**Fig 3:**
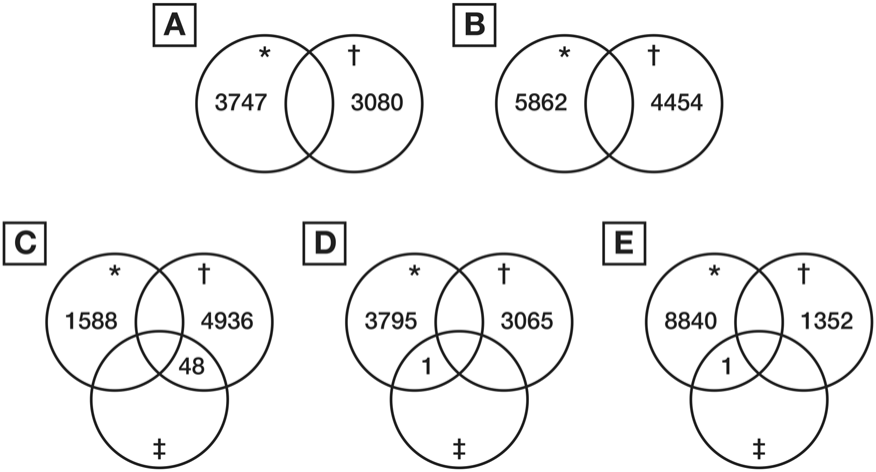
Venn diagram detailing the number of differentially expressed peptides in RIPC as compared to control samples at each time point. **A.** Baseline. **B.** End CPB. **C.** 6 hours post CPB sample. **D.** 12 hours post CPB. **E.** 24 h post CPB. *—down-regulated, †**—**up-regulated, ‡**—**significantly differentially expressed.

The results of the comparison between the groups at each time point are shown as Venn diagrams in Fig. 3. The results demonstrated that there were no peptides with significant differential expression between the RIPC and control groups at baseline or immediately after CPB. At 6 h after CPB, 48 peptides were significantly differentially expressed with all 48 peptides being up regulated in the RIPC group. The response was less pronounced at 12 and 24 h after CPB, with only one peptide down regulated in the RIPC group compared to the control group at each time point.

**Fig 4:**
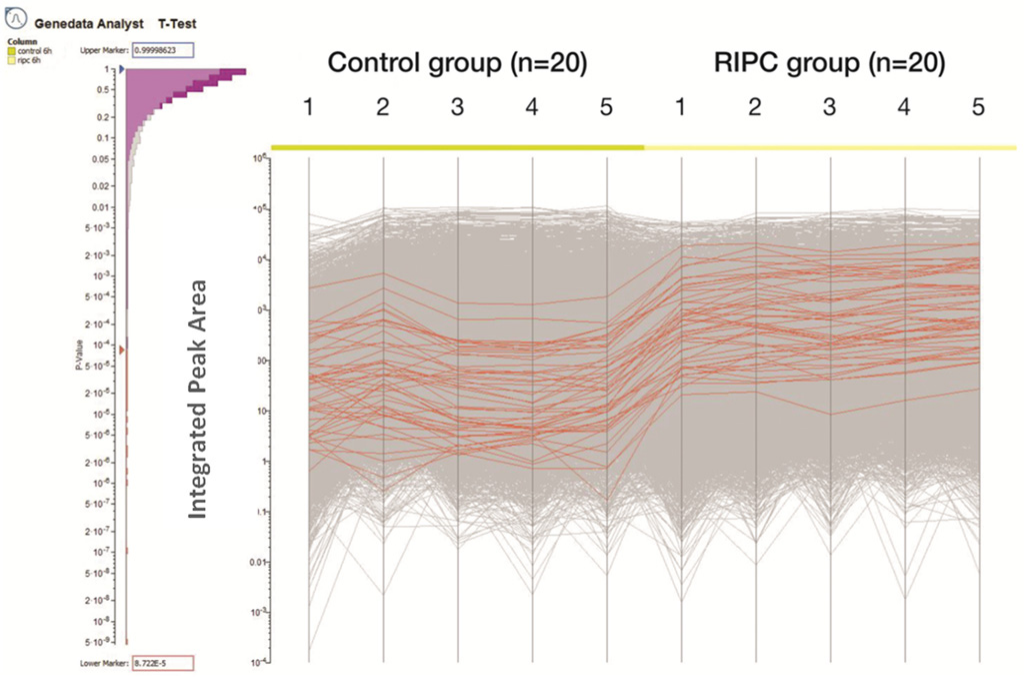
Integrated peak area of the peptides in the 5 pooled samples in each group. Expression of each peptide is depicted as a line with the lines in red each representing the significantly higher expressed peptides in the RIPC group compared to the control group. There were 48 up-regulated peptides in the RIPC group compared to the control group. The histogram on the left indicates the false discovery rate.

The expression patterns for 48 peptides found to be up-regulated in the RIPC group at the 6 h time point are shown in Fig. 4.

These 48 peptides were matched to 6 proteins in the NCBI database as detailed in Table 2. At 12 h post CPB and 24 h post CPB there was one down-regulated peptide, however we were unable to match these peptides to proteins with sufficient confidence.

**Table 2:** Protein data for the 6 proteins that were up-regulated in RIPC group as compared to the control group at 6 hours after CPB.

Protein	Accession number	MASCOT Score	Peak integration area Control	**Peak integration area RIPC**	**p value**
Inter-alpha globulin inhibitor	55958063	9896.84	42.0 ± 11.8	820.8 ± 181.1	0.006
Fibrinogen preproprotein	11761629	22554	59.3 ± 11.2	1192.6 ± 278.3	0.007
Complement C3 precursor	115298678	52035	391.2 ± 160.9	5385.1 ± 689.4	0.0005
Complement C4B	310125237	76.5	151.5 ± 17.8	4587.8 ± 799.2	0.003
Apolipoprotein B100	306569733	542.04	53.4 ± 8.3	1364.5 ± 278.2	0.005
Urinary proteinase inhibitor	262428	45	358.6 ± 74.9	5758.1 ± 1343.1	0.009

**Fig 5:**
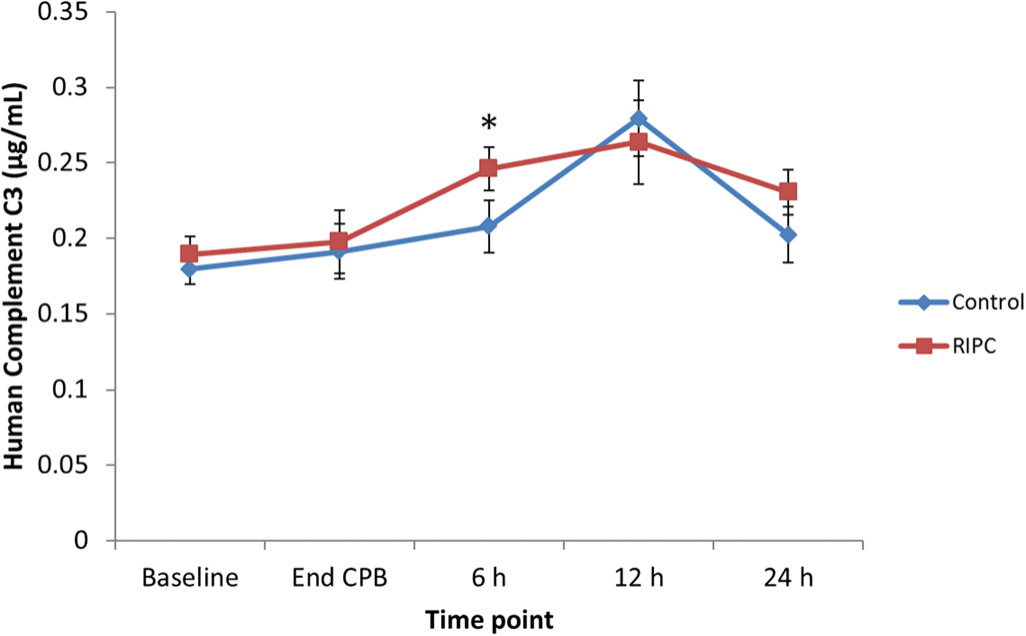
Complement C3 concentration for the control and RIPC groups at the time points in the study. Individual samples were assessed in this ELISA. There was significantly higher levels of Complement C3 in the RIPC group at 6 h after CPB (p<0.05).

The results of the validation study for human Complement C3 (Fig. 5) confirmed significantly higher Complement C3 in the RIPC group at 6 h after CPB (p = 0.048). No difference was found between the groups at any other time point.

## Discussion

RIPC is an intriguing phenomenon that has been applied successfully across a range of clinical settings. Although the mechanisms through which RIPC confers protection have not been fully elucidated, there is evidence that RIPC induces circulating humoral mediators [16,17]. We have previously demonstrated that RIPC induces global genomic changes in healthy adult volunteers and in children undergoing heart surgery [18,19]. We have also previously shown that RIPC modifies the proteomic profile in the plasma of healthy adult volunteers and gained the first clinical insights into the proteins that may be altered during the RIPC stimulus and at 15 min and 24 h after applying the RIPC stimulus [13]. We therefore extended this concept into the clinical setting to test the hypothesis that RIPC modifies the plasma proteomic response in children undergoing repair of Tetralogy of Fallot (TOF). This is the first study to describe the global proteomic response to RIPC in the plasma of children with congenital heart disease undergoing heart surgery. We have identified potential candidate proteins involved in the RIPC mechanism that require confirmation with further studies.

There were no differences in clinical outcomes between the RIPC group and the control group [10], which may be attributed to the fact that chronic hypoxia may already activate the pathways involved in RIPC, so that further augmentation is not possible. This finding is supported by evidence suggesting that RIPC has little effect on the phosphorylation levels of pro-survival protein pathways that are involved in RIPC from the resected right ventricular outflow tract tissue of these patients [10]. Our results are in contrast to studies on the effect of ischemic post conditioning via invasive aortic clamping and de-clamping following reperfusion which showed clinical benefit including decreased morbidity, reduced duration of mechanical ventilation, intensive care unit stay and inotrope requirement in patients undergoing ToF repair [20–22]. Thus, clinical application of the RIPC and its variants still remains a controversial issue and further mechanistic insights are required.

In the current study, the most pronounced proteomic changes between control and RIPC groups occurred at 6 hours after CPB. The 48 peptides with significant higher levels in the RIPC group at 6 hours after CPB were matched to 6 proteins. Four of these 6 proteins were previously found to be differentially expressed in our study of the effect of the RIPC stimulus in healthy adult volunteers [13]. These 4 proteins were inter-alpha globulin inhibitor, fibrinogen preproprotein, complement C4B and apolipoprotein B. These findings consistent with our previous study suggest that there is a common mechanism of protection by RIPC. How these proteins could be linked to the RIPC specific protection is beyond the scope of this study and warrants further investigation. The functions of these proteins have previously been described and are as follows.

The protease inhibitors in blood control proteolysis during complement activation, coagulation, fibrinolysis and inflammation [23]. Inter-alpha globulin inhibitor is an acute phase protein known to be involved in inflammation. It increases in concentration in response to tissue injury. We found up-regulation of inter alpha globulin inhibitor during the RIPC and at 15 min after the RIPC and it is down-regulation at 24 h after the RIPC in our previous study in healthy adult volunteers [13]. This finding of early up-regulation has since been confirmed by Pang *et al*. [24] who reported a greater than 2-fold increase at 1 hour post RIPC stimulus in 60 healthy adult volunteers. In the current study, we found increased levels of inter-alpha globulin inhibitor at 6 h after the CPB, but not at the 12 h or 24 h after CPB. These findings suggested that the inter-alpha globulin inhibitor is involved in the early response to the RIPC stimulus, but returns to the baseline within the first 24 hours. Inter-alpha globulin inhibitor regulates activation and adhesion of leucocytes during inflammation and it also has protease inhibitory activity [25]. Inter-alpha globulin inhibitor is also known to render tissue more resistant to damage from oxygen derived free radicals [26]. Higher levels in the RIPC group may indicate that RIPC-induced protection against oxidative stress.

Fibrinogen preproprotein has previously been demonstrated as being anti-inflammatory in local ischemic preconditioning during acute pancreatitis in rats [27]. The cleavage products of fibrinogen regulate cell adhesion and migration, have vasoconstrictor and chemotactic activities and are mitogens for several cell types [27]. Increased levels of fibrinogen preproprotein at 6 h post CPB may imply decreased cleavage products of fibrinogen as there were higher levels of its precursor. Thus, higher levels of fibrinogen may reflect decreased plasma protease activity and, as such, decreased activation of leucocytes. Furthermore, preconditioning ischemia attenuates platelet activation and aggregation characterized by a decrease in platelets aggregating with fibrinogen [28]. Thus, an anti-inflammatory property of fibrinogen preproprotein in clinical surgery deserves further investigation.

Similarly, the complement C3 precursor was found to be up-regulated at 6 h after CPB. This was confirmed by ELISA. In our preclinical study, we found complement C3 to be up regulated at 15 min post RIPC stimulus [13]. Complement factors play a key role in the inflammatory process. When cleaved, complement C3 precursor causes contraction of smooth muscle, increases vascular permeability and chemotaxis and causes histamine release from mast cells and basophilic leukocytes [29,30]. Complement C3 precursor has been implicated in a number of cellular pathways including the phosphoinositide 3-kinase (PI3K), protein kinase C (PKC), protein kinase B (Akt) and mitogen activated protein kinase /extracellular signal- regulated kinases (MAPK/ERK) pathways, which have also been implicated in RIPC [31].

Complement C4B is important in controlling inflammation, where deficiency and low serum levels are associated with increased susceptibility to inflammation, including systemic lupus erythematosus [32,33]. Complement C4B has anti-inflammatory properties [34]. Increased levels of this protein in the RIPC group 6 h after CPB suggest that RIPC could have an anti-inflammatory effect. This suggests a role for involvement of the complement system in response to RIPC.

Apolipoprotein B levels were high in the RIPC group at 6 hours after CPB in the current study. The precise role of Apolipoprotein B in RIPC is unknown. We have shown apolipoprotein expression to be increased in adults in response to the RIPC stimulus [13]. Furthermore, subsequent studies have confirmed the upregulation of apolipoproteins in response to RIPC in an animal model [35] and have also shown that administration of apolipoprotein prior to prolonged coronary artery occlusion resulted in decreased myocardial infarct area that was as effective as RIPC [36].

Finally, increased level of urinary proteinase in the RIPC groups was an interesting observation. These inter alpha globulin inhibitors are degraded by neutrophils to form urinary proteinase inhibitors [37]. Urinary proteinase inhibitor confers anti-inflammatory effects by decreasing expression of pro-inflammatory cytokines, including TNF-α and interleukin 6, and chemokines [38]. The urinary proteinase inhibitors decrease lipopolysaccharide induced neutrophil activation [38] and have been used therapeutically for patients with inflammatory disease [39]. Administration of exogenous urinary proteinase inhibitors warrants testing in an animal model.

This is the first examination of RIPC induced proteomic changes in the plasma of children with cyanotic heart defects. The results of this study indicate that the RIPC stimulus in children undergoing ToF repair evokes a global proteomic response that peaks at 6 h post-CPB and returns to baseline within the first 24 h. This study provides preliminary insights into the proteins involved in RIPC mediated cellular protection. The observed proteomic changes are consistent with our previous observation in healthy volunteers and may play an integral role in the response to RIPC.

### Limitations

Removal of albumin and Ig-G from plasma allowed for a more thorough analysis of the remaining proteins. This approach did, however, come at a sacrifice as other proteins with an isoelectric point between 5.2 and 6.8 were excluded from the analysis. There may be proteins that we excluded from our analysis that may be important in RIPC associated mechanisms that were not analysed in our study due to the fractionation process that we used.

This study was limited to measuring relative changes in the levels of peptides between the two groups at the various time points and as such involved denaturation and trypsin digestion of the proteins into peptides, therefore, we were unable to assess post translational modifications such as phosphorylation.

Anaesthetic drugs may impact on RIPC responses [40,41], and differentiating the impact of the drugs from RIPC can only be achieved through RCT’s in which the anaesthetic techniques are identical and the only variable is the presence or absence of RIPC. Whether the RIPC leads to clinically important improvements in outcome, will then need to be considered across the range of surgeries and anaesthetics. This study was not powered to detect differences in echocardiogram assessment of diastolic dysfunction.

## Conclusion

We provided the first comprehensive analysis of RIPC-induced proteomic changes in children undergoing heart surgery. The proteomic changes peak at 6 h post-CPB and return to baseline within the first 24 h of surgery.

## Supporting Information

S1 CONSORT Checklist CONSORT Checklist. (DOC)

S1 Protocol Trial Protocol. (DOC)
