# Supplementary material for: Remote Ischemic Preconditioning (RIPC) Modifies the Plasma Proteome in Children Undergoing Repair of Tetralogy of Fallot: A Randomized Controlled Trial
Source: PLoS One. 2015 Mar 31;10(3):e0122778. doi: 10.1371/journal.pone.0122778 (PMC4380409; doi:10.1371/journal.pone.0122778)
Supplement: S1 Protocol — (DOC) [file pone.0122778.s002.doc]

**29133**

**The protective effect of remote ischaemic preconditioning in congenital heart disease patients undergoing cardiopulmonary bypass surgery**

**[A non-invasive way to protect against the stress of surgery for heart disease]**

**VERSION 3, 30/07/2010**

**CONFIDENTIAL**

This document is confidential and the property of Drs. Konstantinov, Cheung, Pepe & Ignjatovic. No part of it may be transmitted, reproduced, published, or used without prior written authorization from the authors.

# STATEMENT OF COMPLIANCE

This document is a protocol for a clinical research study. The study will be conducted in compliance with all stipulations of this protocol, the conditions of ethics committee approval, the NHMRC National Statement on Ethical Conduct in Human Research (2007) and the Note for Guidance on Good Clinical Practice (CPMP/ICH-135/95).

# TABLE OF CONTENTS

Page

1. INVESTIGATORS AND FACILITIES ......................................................................................... 5
   1. Study Location/s ..................................................................................................... 5
   2. Principal Investigators................................................................................................... 5
   3. Funding and resources ............................................................................................... 5
2. INTRODUCTION AND BACKGROUND .................................................................................... 6
   1. Background Information ............................................................................................ 6
   2. Research Question ..................................................................................................... 8
   3. Rationale for Current Study ................................................................................... 8
3. STUDY OBJECTIVES .............................................................................................................. 8
   1. Primary Objective ..................................................................................................... 8
   2. Secondary Objectives ................................................................................................ 9
4. STUDY DESIGN ................................................................................................................... 9
   1. Type of Study .................................. ...................................................................... 9
   2. Number of Subjects ................................................................................................... 9
   3. Expected Duration of Study .................................................................................... 9
   4. Primary and Secondary Outcome Measures ............................................................. 9
5. STUDY TREATMENTS ........................................................................................................ 10

5.1 Treatment Arms ................................................................................................... 10

5.1.1 Description ................................................................................................... 10

5.1.2 Dosage and Route of Administration ................................................................ 10

5.1.3 Heart Surgery ..................................................................................................... 10

5.1.4 Myocardial Sampling............................................................................................... 10

5.1.5 Blood Sampling..................................................................................................... 10

1. SUBJECT ENROLLMENT AND RANDOMISATION ................................................................... 11
   1. Recruitment ......................................................................................................... 11
   2. Eligibility Criteria ....................................................................................................... 11
      1. Inclusion Criteria ..................................................................................... 11
      2. Exclusion Criteria .................................................................................... 11
   3. Randomisation Procedures ................................................................................. 11
   4. Blinding Arrangements ................................................................... ......................... 11
   5. Breaking of the Study Blind .................................................................................. 11
   6. Subject Withdrawal ................................ .................................................................. 12
      1. Reasons for withdrawal .................................. ............................................ 12
      2. Replacements ............................................................................................. 12
   7. Trial Closure .............................................................................................................. 12
2. STUDY VISIT AND PROCEDURE SCHEDULE .............................................................................. 12
3. CLINICAL AND LABORATORY ASSESSMENTS ........................................................................... 12
4. ADVERSE EVENT REPORTING .................................................................................................. 16

9.1 Definitions ............................................................................................................. 16

9.2 Assessment and Documentation of Adverse Events .............................................. 16

9.3 Serious Adverse Event Reporting .......................................................................... 16

1. STATISTICAL METHODS ..................................................................................................... 17

10.1 Sample Size Estimation ......................................................................................... 17

10.2 Population to be analysed .................................................................................... 17

10.3 Statistical Analysis Plan ......................................................................................... 17

10.4 Interim Analyses .................................................................................................. 17

1. DATA MANAGEMENT .......................................................................................................... 17

11.1 Data Collection ................................................................................................... 17

11.2 Data Storage ....................................................................................................... 17

11.3 Study Record Retention ...................................................................................... 17

1. ADMINISTRATIVE ASPECTS ................................................................................................... 18
   1. Confidentiality ..................................................................................................... 18

12.2 Participant Reimbursement ................................................................................... 18

12.3 Financial Disclosure and Conflicts of Interest ............................................................ 18

1. USE OF DATA AND PUBLICATIONS POLICY ........................................................................... 18
2. TRIAL REGISTRATION ……………………………………………………………………………………………………….. 18
3. REFERENCES ..................................................................................................................... 18
4. APPENDIX ......................................................................................................................... 22

# PROTOCOL SYNOPSIS

| Title | The protective effect of remote ischaemic preconditioning in congenital heart disease patients undergoing cardiopulmonary bypass surgery |
| --- | --- |
| Objectives | Support of the circulation during heart surgery using the heart-lung bypass machine is inevitably associated with organ damage and associated reduced function. This is due to reduced blood flow (ischaemia), the effects of restoration of flow (reperfusion injury) and the subsequent inflammation that is caused. The body has its own way of protecting itself against reduced blood flow and oxygen by a mechanism known as preconditioning. In essence, brief periods of mild ischaemia are protective against a subsequent more severe episode of ischaemia. These periods of mild ischaemia can be of the organ itself or of another organ in the body. For example ischaemia of the leg can protect the heart against ischaemia, so called “remote preconditioning”. We have shown in animal and human models that remote preconditioning using a tourniquet placed around a thigh for brief periods (similar in duration to when taking blood samples from children) reduces the amount of injury to heart muscle by 50% and also leads to improved heart and lung function. We have shown that remote preconditioning in a similar way protects the organs of a heterogeneous group of children undergoing cardiac surgery, resulting in better function of the heart and lungs and also a reduction of the inflammatory response to the heart-lung machine. This could potentially reduce the problems in looking after children after surgery and also reduce the amount of time spent in the intensive care unit.  We will study a more uniform group of patients undergoing cardiac surgery in the neonatal period inorder to identify key protein and metabolic changes that occur within this cardioprotective protocol. All interventions will be performed during the period of routine general anaesthesia at the time of surgical repair. We will study the degree of organ injury induced by heart-lung bypass using standard intensive care parameters and equipment for measuring lung function. The degree of heart muscle and blood cell metabolic function will be assessed by laboratory tests. Samples will be taken from routinely discarded resected cardiac tissue and indwelling catheters routinely placed at the time of surgery and not require additional venepuncture. Measurements will be made prior to surgery and also at set time intervals in the first 24 hours postoperatively to determine the evolution of effects. |

# GLOSSARY OF ABBREVIATIONS

| **ABBREVIATION** | **TERM** |
| --- | --- |
| CPB | cardiopulmonary bypass |
| IR | ischemia-reperfusion |
| IPC | ischemic preconditioning |
| MI | myocardial infarction |
| RIPC | remote ischemic preconditioning |
| mmHg | millimetres of mercury, unit of blood pressure |
| CICU | cardiac intensive care unit |
| M | molar (moles/litre) |
| µM | micromolar |
| µl | microlitres |
| U | units |
| mg | milligrams |
| kg | kilograms |
| µg | micrograms |
| m^2^ | metre squared (unit of area) |
| O_2_ | oxygen |
| DIA | Differential In-gel analysis |
| nm | nanometre |
| ε | Extinction coefficient |
| MPTP | Mitochondrial membrane permeability transition pore |
| MCRI | Murdoch Children’s Research Institute |
| RCH | Royal Children’s Hospital |
|  |  |
|  |  |
|  |  |
|  |  |
|  |  |
|  |  |
|  |  |
|  |  |
|  |  |

# INVESTIGATORS AND FACILITIES

- 1. **Study Location/s**

Royal Children’s Hospital: Departments of Cardiac Surgery and Cardiology; and Murdoch Children’s Research Institute: Heart Research and Haematology Research

- 1. **Principal Investigators**

Prof. Igor Konstantinov, Dr Michael Cheung, Dr Salvatore Pepe, Dr Vera Ignjatovic, Michele Hepponstall

- 1. **Funding and resources**

National Health and Medical Research Council Project Grants 607404 and 628756

# INTRODUCTION AND BACKGROUND

- 1. **Background Information**

Patients undergoing heart surgery with standard cardiac arrest and cardiopulmonary bypass (CPB) develop tissue injury due to myocardial ischemia and systemic inflammatory response to CPB. Paradoxically, most of the myocardial damage occurs once the coronary artery is reopened and the blood flow is restored. This phenomenon is referred to as an ischemia-reperfusion (IR) injury and associated with a systemic inflammatory response, myocardial dysfunction, and cardiac arrhythmia. Furthermore, systemic inflammatory response induces post-operative multi-organ dysfunction of various degrees that results in prolonged hospital stay and delayed recovery. Protection against IR injury is of particular importance in children undergoing heart surgery as, due the severity of heart anomalies, it often requires prolonged period of heart arrest to perform a complex repair. Such protection is also important in adult patients with decreased heart function due to recent myocardial infarction undergoing heart surgery or angioplasty.

One of the most fascinating physiological mechanisms of nature is the innate mechanism by which all living cells protect themselves from the lack or excess of oxygen that occur during IR injury. This powerful innate protection can be induced by a brief period of ischemia that precedes a prolonged, life-threatening period of ischemia. This phenomenon is known as ischemic preconditioning (IPC). The IPC reduces myocardial infarction (MI) by 60% following coronary artery occlusion (1,2) and has been shown to reduce myocardial injury following heart surgery (3). In addition to reducing IR injury, the IPC also modulates activation of inflammatory cells that are induced by the IR injury. In a novel human model, we previously showed that IPC not only prevents endothelial dysfunction but also significantly attenuates the secondary activation and inflammatory gene expression of circulating leukocytes (4, 5).

A more practical and clinically relevant form of protection is remote IPC where ischemia of one organ can induce a protected state in a distant organ (6). The magnitude of the effect of remote IPC appears to approach that of local IPC. A reduction in MI size of 65% was reported in a porcine model study by inducing ischemia of the lower limb (7). As with local IPC, the mediators and mechanisms of remote IPC remain to be fully elucidated but presumably include humoral agents as well as induction of gene expression (8-10).


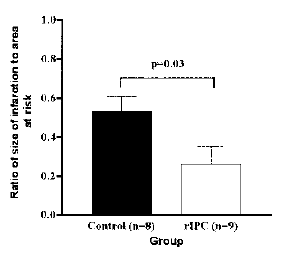
We have previously demonstrated in an animal model of MI, the potential of remote IPC to reduce myocardial IR injury (11, 12). These studies demonstrated a 50% reduction in the extent of MI in the group with prior remote IPC compared to the control group (Figure 1). Remarkably, a brief limb ischemia applied during evolving MI in pigs resulted in similar reduction of MI (12). Furthermore, in an animal model of remote IPC and CPB we have shown significantly lower levels of plasma lactate, myocardial troponins and S-100B (markers of cardiac and brain injury respectively) (Figure 2), increased dynamic lung compliance, better cardiac diastolic function and a lesser requirement for inotropes in study animals as compared to controls (13,14). In addition, despite 2 hours of warm heart ischemia, none of the preconditioned animals required inotropes to wean from CPB (all controls did).

FIG 2. (From Kharbanda RK, et al. Heart 2006 Oct; 92(10):1506-11).

We have previously demonstrated in a porcine model that myocardium contributes significantly to total metabolic burden following hypothermic CPB (23) and that remote IPC significant decrease in coronary resistance and improved myocardial blood flow (15). After completion of our animal experiments, we applied the remote IPC concept in clinical practice and published world’s first clinical randomized controlled study of the remote IPC in children undergoing cardiac surgery that demonstrated significant cardio-pulmonary protection, particularly with regard to reduced post-CPB release of troponin I, lactate, S100B and reduced requirement for post-operative inotropes (16, 17). Subsequently, others confirmed protective effect of the remote IPC in patients undergoing coronary artery bypass surgery (18) and significant reduction in myocardial and renal injury after abdominal aortic aneurysm repair (30). Due to complexity of congenital heart defects, heart surgery in children is often associated with prolonged IR and CPB. This, in turn, results in extensive IR injury and systemic inflammatory response. A reproducible and powerful induction of innate protection against IR injury and CPB-induced systemic inflammatory response would be of particular benefit in this subgroup of patients. Although clinical benefits of protection by the remote IPC became apparent in the recent clinical trials (16-19, 30), the mechanisms for this clinically relevant protection remain yet to be elucidated (19-21).

From our previous work in a porcine heart transplant model significant myocardial protection could be achieved in the donor heart following transplantation into a preconditioned donor (9). The study ruled out the neurogenic mechanism of protection, as the transplanted heart was denervated, and suggested that remote IPC produces a blood-borne protective factor (9). This study also demonstrated that remote IPC induced a circulating factor in the blood that provided myocardial protection in transplanted heart. In other words, the remote IPC induced a lasting protected environment in the recipient prior to transplantation of the donor heart (i.e., the heart was not in the body during the IPC stimulus). This study suggested the presence of a humoral circulating factor in blood. We then further localized a “protective factor” and demonstrated in an animal model that remote IPC elaborated a transferable plasma-borne factor that protected mitochondrial structure and function and preserved myocardial performance following neonatal cardioplegic arrest (22). This metabolic and structural protection was associated with preservation of global myocardial performance (22). We have further studied human leukocyte functional responses in our pilot study performed in healthy human volunteers (24) and demonstrated that the remote IPC stimulus significantly decreased neutrophil adhesion, exocytosis, and phagocytosis. Our human pilot study (24) as well as our global genomic study (5) suggested that proteomic response to remote IPC stimulus will be significant. Others have also demonstrated that IPC can be abolished by inhibition of de novo protein synthesis at both transcription and translation levels (31-32). Furthermore, our previous genomic studies demonstrated significant changes in global gene expression in murine myocardium after the remote IPC stimulus (10) and in the human myocardium in children undergoing heart surgery (25). However, it remains unknown if the global genomic response observed in our previous studies results in translation of expressed genes into proteins. Over the recent year proteomic profiling has evolved as a reliable, reproducible and powerful technique of assessment of global protein expression in response to clinically relevant IR injury (33,34) and particularly CPB (35). This technique allows simultaneous assessment of expression of a great number of thematically linked proteins, including mitochondrial metabolic signalling proteins that may constitute protective pathways.

- 1. **Research Question**

Based on the above background and our previous studies, we will endeavour to identify a plasma-borne factor produced by brief repetitive episodes of ischemia-reperfusion (IPC) in the thigh that induces a protective state in remote organs (remote ischemic preconditioning, RIPC). We will assess the global proteomic and mitochondrial metabolic response to IR injury in children undergoing heart surgery with CPB and the modification of such a proteomic response induced by remote IPC stimulus. Proteomic assessment and mitochondrial metabolism methodology will be employed concurrently.

- 1. **Rationale for Current Study**

We hypothesise that brief repetitive episodes of ischemia and reperfusion in the thigh release a plasma-borne factor that induces a protective state in remote organs and significantly modifies systemic inflammatory response to IR injury and modulates mitochondrial metabolic function in heart muscle and lymphocytes. These effects may afford augmented organ protection to neonatal children undergoing cardiopulmonary bypass surgery. Characterization and identification of the remote IPC protective factor and pathways that foster recruitment of innate protective mechanisms against organ dysfunction following IR injury will be crucial to tailoring more efficient and clinically practical protective therapy. The future clinical application of such protection could be immense and may ultimately encompass organ transplantation, protection against stroke, myocardial infarction, acute multi-organ dysfunction, and attenuation of systemic inflammatory response in diverse clinical scenarios.

# STUDY OBJECTIVES

- 1. **Primary Objective**

Does remote IPC modify the global plasma proteomic and mitochondrial response in patients undergoing congenital heart surgery?

**Hypothesis:**

Remote IPC induces significant changes to global proteomic response in plasma and the metabolic function in heart muscle and lymphocyte mitochondria of patients undergoing congenital heart surgery.

**Aim:**

To assess the impact of remote IPC on the global proteomic and mitochondrial metabolic function response to heart surgery with CPB support.

- 1. **Secondary Objectives**

Does remote IPC modify clinical markers of injury and cardiopulmonary function post-CPB in patients undergoing congenital heart surgery?

**Hypothesis:**

Remote IPC reduces post-CPB release of markers of cellular injury and permits recovery of cardiac pump function with less complication including inotrope requirement in patients undergoing congenital heart surgery.

**Aim:**

To assess the impact of remote IPC on lactate release, inotrope requirement, and standard clinical cardiopulmonary assessments in response to heart surgery with CPB support.

# STUDY DESIGN

- 1. **Type of Study**

Small randomised, blinded, surgical trial of preoperative therapy with highly regulated, brief, non-invasively-induced, localised leg ischaemia and reperfusion, for its capacity to invoke remote ischemic preconditioning-related molecular signalling in blood and heart muscle.

- 1. **Number of Subjects**

N=40 Total.

- 1. **Expected Duration of Study**

March 2010-March2012

- 1. **Primary and Secondary Outcome Measures**

**Primary outcome measures:** The identification of molecular signalling in blood and heart muscle in response to RIPC induced by highly regulated, non-invasively-induced, localised leg ischaemia (5min) and reperfusion (5min) for 4 cycles.

**Secondary outcome measures:**

Alveolar-arterial (A-a) oxygen gradient and lung compliance will be obtained as a part of routine clinical assessment. Measures of lung function oxygenation index and alveolar-arterial oxygen gradient at 3, 6, 12, 24 hours post-op. Measurements including mean systemic arterial, and right atrial pressures are made at 3, 6, 12, and 24 hours after aortic cross clamp release. The doses of inotropic, chronotropic, and afterload reducing agents at the time of the cardiac output measurements are recorded. For between-group comparisons, a total inotrope dose score is calculated by adding the doses of dopamine and dobutamine in micrograms per kilogram per minute and assigning an arbitrary equivalent value of 10µg•kg-1•min-1 inotrope for each 0.1µg•kg-1•min-1 epinephrine. Daily post-operative measurements of arterial blood gases, serum glucose, calcium, electrolytes, hematologic parameters, arterial lactate, blood urea nitrogen, and creatinine, the total volume and type of fluid intake, urine output, and chest tube drainage, the total hours of mechanical ventilation and the days in the CICU and the hospital are recorded.

# STUDY TREATMENTS

- 1. **Treatment Arms**

**5.1.1 Description**

The hypothesis will be tested in children with tetralogy of Fallot undergoing elective open heart surgery with standard heart arrest and cardiopulmonary bypass. An informed consent will be obtained from all the patients’ parents or guardians. 40 patients will be recruited in to the study within two years. They will be randomized into 2 equal sized groups: Group I control (Sham, n=20) and Group II preconditioning (RIPC, n=20).

**5.1.2 Dosage and Route of Administration: RIPC Protocol**

RIPC will be instituted by four 5-minute cycles of leg ischemia with intervening 5 minutes of reperfusion immediately following induction of standard anaesthesia in the operating room. A standard WelchAllen blood pressure cuff is placed on the mid-to upper thigh of the leg which is free of i.v./arterial lines. Inflating the blood pressure cuff to a pressure exceeding the patient’s systolic pressure by 30 mmHg will constrict the site to interrupt local blood flow and thus perfusion of the lower leg (i.e. if systolic pressure is 70mmHg then the cuff will be inflated to 100mmHg). The cuff would be deflated to permit reperfusion. Blood flow interruption and restoration will be monitored by standard pulse-oxymetry. Control patients will have sham placement of cuff without inflation. RIPC will be performed after the patient has been anesthetised while routine central lines are being placed. The protocol is safe and has been previously applied in children by us without any complications or adverse consequences as we have previously published and as is the case with our study in progress at the RCH (HREC#26091C).

**5.1.3 Heart surgery**

Anaesthetics and bypass protocol will not change from the usual practice within the hospital. Open heart surgery for tetralogy of Fallot will be performed in a standard fashion with cardioplegic heart arrest and cardiopulmonary bypass. Our group has demonstrated expertise in studying patients undergoing heart surgery (17, 22, 25-29).

**5.1.4 Myocardial sampling**

Muscular obstruction of the right ventricular outflow tract will be resected in routine fashion following administration of standard cardioplegic solution. Resected right ventricular myocardium that is routinely discarded (approximately 10-15mg – pea size) will be saved and processed for mitochondrial function and histological measures. As per our previously published studies in children undergoing resection of the right ventricular outflow obstruction, myocardial samples are immediately transferred on ice for laboratory assays (24, 27).

**5.1.5 Blood sampling**

The following 5 blood samples (6.5 ml each) will be taken from the arterial line that is routinely placed in all patients: baseline, immediately after discontinuation of CPB, at 6, 12, and 24 hours following discontinuation of CPB. No additional lines or invasive procedures will be performed apart from those that routinely required for heart surgery. From each sample, 1.5 ml will be used for proteomic assays, and 5 ml for the lymphocyte mitochondrial function assays.

# SUBJECT ENROLLMENT AND RANDOMISATION

- 1. **Recruitment**

Patients will be recruited by either the surgeon (Konstantinov) or cardiologist (Cheung) at the preadmission clinic or on the ward prior to surgery. The clinician (Konstantinov/Cheung) will explain to the patient’s parents/guardians, the research project, their child’s role, and outcomes and will obtain signed consent if granted.

- 1. **Eligibility Criteria**
     1. **Inclusion Criteria**

Patients with tetralogy of Fallot aged one month and older (to 18 years) having surgery for the first time.

- - 1. **Exclusion Criteria**

Patients with chromosomal defects, associated congenital lung malformations and haematological disorders will be excluded.

- 1. **Randomisation Procedures**

Random allocation is made in blocks in order to keep the sizes of the 2 treatment groups similar. In order to avoid predictability, the block size that is randomly selected is 2, 4 or 5, as the sample size must be divisible by the block size. Due to the expected relative uniformity of condition in the patient group and the small number of subjects the design will not be stratified. The randomisation will be performed in secret by a member from Heart Research (not involved in working for the trial) using SPSS statistical software generated randomisation and will place each designation sequentially in numbered envelopes 1 to 40, for patient 1 to 40 and the envelopes will be signed and sealed. The implementer of the RIPC or Sham co ntrol treatment protocols will open one envelope in numerical order immediately prior to placement of the blood pressure cuff and they will apply the RIPC treatment or sham control protocol accordingly.

- 1. **Blinding Arrangements**

Ms Hepponstall will not be blinded as she will be applying the RIPC treatment. She will also be involved in randomisation, sample collection, data collection, data entry and laboratory analysis. Surgeons, perfusionists, anaesthetists will be blinded. Samples will be de-identified and therefore, research laboratory staff will be blinded to the patient’s identity. The blood pressure cuff will be positioned on the thigh for both sham control and RIPC groups, however if other theatre staff witness whether the RIPC treatment protocol is invoked they will not be blinded. As these theatre staff are strictly performing standard clinical tasks and procedures and are not involved in the trial and analysis *per se*, this should not impact the blinding.

- 1. **Breaking of the Study Blind**

This will occur on completion of the final statistical analyses, and will be performed in the presence of the independent professional statistician (see 10.3).

- 1. **Subject Withdrawal**
     1. **Reasons for withdrawal**

No reason needs to be made. Only a request for withdrawal needs to be made by the subjects’ parents or guardian, (ideally prior to surgery) to Dr. Cheung or Prof. Konstantinov. It is unlikely that a parent will withdraw the subject following surgery.

- - 1. **Replacements**

In the event of a withdrawal in which the subject has not had surgery (and thus not been treated) additional recruitment will continue until n=40 subjects are completed.

- 1. **Trial Closure**

This will occur upon completion of the 40th subject.

# STUDY VISITS AND PROCEDURES SCHEDULE

The subjects are directly studied only during the perioperative period. Blood and myocardial samples are studied upon arrival to the laboratory (mitochondrial studies) or in batches after freezing (proteomic/microscopy studies).

# CLINICAL AND LABORATORY ASSESSMENTS

**8.1 Proteomic Assessment Methodology**

The plasma samples will be analysed as per protocol outlined below in Figure 3: Strategy for proteomic profiling of plasma samples.

**8.1.1. Sample Preparation**

Plasma samples will be depleted of 4 highest abundance proteins (albumin, alpha-antitrypsin, transferring and haptoglobin) that are of little pathological importance, using the ProteoSpin Abundant Serum Protein Depletion Kit (Norgen Biotek) to unmask the presence of lower abundance proteins of interest. The remaining proteins will be precipitated using the acetone precipitation protocol, as specified in the kit. Removal of these proteins increases the sensitivity of detection for proteins of interest. The protein pellets resulting from acetone precipitation will be re-suspended in 50µl DIGE labelling buffer containing 7M urea, 2M thiourea, 4% CHAPS and 30mM Tris, for 3 hours. Protein content of each sample will be quantified using the Bradford assay, using the Protein Assay Dye Reagent Concentrate (Bio-Rad).

**8.1.2. Sample Labelling and 2D-DIGE**

The internal standard consisting of an equal amount of each one of the two samples to be analysed on each gel will be labelled with the Cy2 dye (GE Healthcare), to minimize gel-to-gel variation. For each time-point, 50% samples will be labelled with Cy3 and 50% samples with the Cy5 dye, with the samples then randomized to gels (2 samples per gel).

Sample labelling will be performed according to the recommendations set by GE Healthcare. The Cy2, Cy3 and Cy5 samples for each gel will be pooled and the loading volume adjusted with labelling buffer (specified above) to 100µl prior to loading onto the 1st Dimension IPG Strip. The 24cm, pH3-11 non-linear Immobiline Drystrips (GE Healthcare) will be rehydrated overnight with rehydration buffer (15ul IPG buffer 3-11NL and 3ml DeStreak solution) from GE Healthcare. Isoelectric focusing will be carried out using the Ettan IPGphor3 Isoelectric Focusing system (GE Healthcare). This system includes software that monitors and records parameters for each run, allowing for standardization across runs. The IPG strips will then be conditioned for 15 min in equilibration buffer containing 2%SDS, 50mM Tris-HCl, pH8.8, 6M urea, 30% glycerol, 0.002% bromophenol blue, and 10mg/mL DTT. The strips will then be alkylated for 15 min in equilibration buffer containing 25mg/mL iodoacetamide instead of the DTT and loaded onto 12.5% polyacrylamide gels. Second dimension will be run using the Ettan Dalt 6 system (GE Healthcare), for an average of 4.5 hours, until the bromophenol blue dye-front reaches the edge of the gels.

**8.1.3. Gel Imaging**

Gels will be scanned using the Typhoon 9410 imager (GE Healthcare), with a resolution of 100µm, and PMT of 500/600V.

**8.1.4. Gel Data Analysis**

Data obtained from the gels will be quantified using the DeCyder version 6.5 software (GE Healthcare). The Differential In-gel analysis (DIA) will be used to optimize spot detection, with the master gel being assigned automatically to the gels with most spots detected. The Biological Variation Analysis (BVA) module will be used for analysis of each sample according to the corresponding age-group. The filtering parameters will then be set to determine the spots that: had a p-value ≤ 0.05 for the t-test employed to test the variation between age-groups and a >1.5 fold change in abundance between the groups.

**8.1.5. Spot Excision and Identification**

Proteins of interest (determined by change from baseline) will be excised robotically using the Ettan Spot-picker (GE Healthcare) and identified by tandem mass spectrometry (MS/MS) on an HPLC-Chip ESI-Trap XCT tandem mass spectrometer or LTQ-FT mass spectrometer. Protein identification will be verified with the use of western blots using specific antibodies.

**8.2 Assessment of Mitochondrial Function (myocardial & lymphocyte)**

Ischemia-reperfusion injury diminishes: mito complex I and IV activities, limits maximal O2 consumption capacity and the efficiency of mito O2 consumption, including greater propensity for the loss of mitochondrial membrane potential (36). Thus the strategy to assess evidence for cardioprotection induced by IPC protocol will be to measure the integrity of mitochondrial respiratory chain enzymes; their activity rates as well as the activation of upstream signalling protein kinases. As our laboratory has long experience in miniaturised metabolic assays for very small clinical samples of tissue and blood (with aid of highly sensitive Cary 300 and Nanodrop spectrophotometers) a number of concurrent assays are permissible in each sample.

**8.2 .1. Mitochondrial Isolation & Preparation**

Mitochondria are isolated from resected myocardial tissue using our custom protocols that permit viable yields from myocardium weighing 5mg or more. In addition, lymphocytes are isolated from whole blood (5ml) by Ficoll-Paque density centrifugation which yields approximately 3-10x106cells/ml. While some lymphocytes are set aside for IPC signalling protein assessments, for mitochondrial respirometric experiments, lymphocytes are permeabilised with digitonin (100 μg/mL) for 5 min in the presence of protease inhibitor mixture (Roche) 1mM 4-(2-aminoethyl)-benzenesulphonyl-fluoride hydrochloride (Roche) and 2mM diisopropyl fluorophosphate (Fluka), in a buffer of 250ml sucrose, 10mM HEPES pH7.4 and 1mM EDTA and then digitonin is washed out via centrifugation (1000xg, 4ºC, 5 min, twice).

**8.2.2. Mitochondrial Oxygen Consumption**

As per previous studies (37-39), State III and IV substrate-coupled mitochondrial O2 consumption is measured via a miniature Clarke-type O2 electrode and closed-volume 30μL glass chamber (37ºC) in pyruvate/malate buffer (2.5mM/0.5mM), driven by 0.5mM ADP. Maximal O2 consumption rate is determined with the proton gradient uncoupler, FCCP (2μM). Complex I-specific O2 consumption is determined with the inhibitor, rotenone (5μM), for complex III, antinomycin A (4μM). Non-mitochondrial sources of O2 consumption are assessed after the addition of 5mM sodium cyanide (complex IV inhibitor). Stoichiometric estimate of ADP:O consumption is used as an index of efficiency of mitochondrial oxygen use.

**8.2.3. Respiration Enzyme Activities**

In vitro activities for complexes I (NADH dehydrogenase), II (succinate dehydrogenase), III cytochrome bc oxidoreductase, IV (cytochrome c oxidase), are assessed on a Cary 300 spectrophotometer equipped with magnetic stirring and thermostatic control. Activities are normalised to total protein levels and the mitochondrial enzyme, citrate synthase. Complex I, II, III & IV assays are performed via spectrophotometry at 340nm (ε=6.81mM-1cm-1); 600nm (ε=19.1mM-1cm-1); 550nm (ε=19mM-1cm-1); 550nm (ε=19mM-1cm-1), respectively. Citrate synthase is measured spectrophotometrically at 412nm for 2mins during its conversion of Acetyl CoA + Oxaloacetate to citrate and CoASH, and compared to standards at 0, 2, 4, 6, 8 and 10 U/ml (Sigma).

**8.2.4. MPTP**

Mitochondrial membrane permeability transition pore (MPTP) opening in vitro precedes the collapse of the proton-motive force, disruption of ionic homeostasis and membrane potential, mitochondrial swelling, and rapid ATP hydrolysis that, when occurring irreversibly, accompanies signalling for apoptotic cell death. The feature of mitochondrial swelling coupled to MPTP open-closed transitions is a useful marker for invitro assessment for propensity for MPTP pore opening which occurs with the dissipation of mitochondrial membrane potential, Δψ. Mitochondrial membrane potential is estimated by following safranin O (5μM) calibrated fluorescence at an excitation wavelength of 495nm and emission wavelength of 586nm, using 5nm slits, on a Perkin Elmer spectrofluorometer(40). In separate experiments calcium-sensitised molecular-triggered volume changes (mitochondrial swelling) of isolated mitochondria (myocardium) or permeabilised lymphocytes that are linked to MPTP opening are measured via spectrophotometric absorbance changes at 540 nm in 30μl. In these studies, the propensity for MPTP opening and the rate of onset will be assessed either in myocardial mitochondria or lymphocyte preparations will be assessed from sham control or RIPC pretreated patient groups. In addition we will investigate the dose-dependant effects of key cytokines such as IL-1, IL-6, TNFα and IL-10 on MPTP opening or blockade of RIPC protective effects.

**8.2.5. Western Blot Analysis of Protein Expression, Phosphorylation, and Translocation**

As IPC has been demonstrated to involve activation of a number of protein kinases involved in cardioprotective signal transduction (40-42), the phosphorylation of the PI3Kinase-dependent proteins Akt at Ser473, GSK-3beta at Ser9 and STAT3 at Tyr705 is assessed in myocardial or lymphocyte samples via SDS-polyacrylamide gel electrophoresis protein blotting with respective monoclonal antibodies (R&D Systems, Santa Cruz Biotechnology, Chemicon and Promega). Extracted protein content is examined via SDS-PAGE (electrophoresis/immunoblot) whereby equal quantities of protein (25 μg) from total or sub-cellular fractions isolated via differential centrifugation (cytosolic, mitochondrial, sarcolemmal membrane) are loaded into gel lanes. Component proteins resolved by SDS-PAGE are then transferred electrophoretically to nitrocellulose membranes. Membranes are blocked, washed and probed with primary antibodies for selected proteins (total and phosphorylated). Washed membranes are incubated with secondary antibody, and immunoreactive bands measure densitometrically on a UVP EC3 ChemiHR410 chemiluminescence imaging system.

**8.2.6. Leukocyte Apoptosis**

Apoptosis is assessed using a combination of propidium iodide and annexin V-FITC (R&D systems, MP) fluorescence staining with quantification by flow cytometry as previously described (43).

**8.2.7. Electron Microscopy**

Heart muscle cut into small cubes (~2mm) or cells attached to slides in calcium-free buffer are initially fixed with 4% paraformaldehyde + 0.05% glutaraldehyde. Sections are incubated with antibodies of interest [i.e. Connexin 43, (localizes to mitochondria in preconditioning) in alternate sections], then with gold-conjugated secondary antibodies (R&D Antibodies), fixed with 2% glutaraldehyde, postfixed with OsO4, dehydrated, and embedded into Epon. Ultrathin sections are viewed with a JEOL electron microscope. In addition to immunogold localization of antibodies, mitochondrial and cellular membranes are screened for integrity or injury.

# ADVERSE EVENT REPORTING

- 1. **Definitions**

Adverse event related to the remote preconditioning stimulus will be reported. A potential local effect is significant vascular compromise of the preconditioned limb although this has not been observed in any of the humans studied to date in current studies (HREC#26091C) or previously published studies. Whether or not an event is a serious adverse event (SAE) is at the discretion of the clinical investigator. For this clinical trial, the following will be considered as severe adverse events and will be reported to the Human Research and Ethics Committee if they occur within 30 days of surgery:

1. Death,

2. Stroke,

3. Permanent disablement,

4. Mechanical support

5. Unplanned re-admission

- 1. **Assessment and Documentation of Adverse Events**

Subjects are closely monitored by responsible theatre staff for well being in the perioperative and post-operative period as by standard medical care. Limb perfusion is monitored daily by the nursing staff in the postoperative period in intensive care. Patients will be followed for the period of their intensive care admission which is typically at least 5 days.

SAE data if occurring, will be collected from medical notes for patients cared for at the Royal Children’s Hospital and by telephone interview of the referring doctors for interstate patients if they have returned to the referring centre prior to 30 days post-surgery.

On each SAE, the following will be reported, as per the RCH reporting form (see Appendix): Date of occurrence; Event details (synopsis); Event management (synopsis); Event outcome (synopsis); Is it:        Expected or Unexpected; Is it:        Unrelated, Possibly related, Probably related or Definitely related.

- 1. **Serious Adverse Event Reporting**

SAE Report Forms (See Appendix) will be forwarded without delay (within 24 hours) to the clinical Principal Investigators (Prof Igor Konstantinov or Dr Cheung), and to the Chair of the RCH Human Research Ethics Committee within 72 hours.

# STATISTICAL METHODS

**10.1 Sample Size Estimation**

Sample size is based primarily on the successful study of RIPC in children (see ref 17) where n=approximately 20 per treatment group. This prior study was amply powered to detect differences in clinical and molecular measures. Notably, post-operative inotrope usage was greatly diminished by RIPC. Based on these inotrope usage results, a power of 0.8, alpha=0.05 and sigma of 3.9 indicates that a sample size of n=20 was required. Injury was deemed to be diminished by RIPC on the basis of reduced troponin I release, thus for a power of 0.8, alpha of 0.05 and a sigma of 3 the sample size required is calculated as n=16. Based on our laboratory’s ability to detect a 20% difference in mitochondrial oxygen consumption in human tissue preparations at least 10 subjects are required to detect this difference (power=0.8, sigma=14.5, alpha=0.05, 2 groups. Similarly, the detection of changes in many mitochondrial proteins measured by proteomic assays, are also quite sensitive ranging between 10-20%. Thus a sample size of n=20 per group will yield ample statistical power.

**10.2 Population to be analysed**

The subjects are recruited and consented tetralogy of Fallot patients undergoing first time corrective surgical palliation.

**10.3 Statistical Analysis Plan**

Lab Assay analyses will be performed on coded samples by Drs Pepe and Ignjatovic and the values then made available for statistical testing when all tests are complete. Statistical analyses will be performed by Drs Konstantinov, Pepe & Ignatovic. An independent professional statistician experienced in trials will be employed to check and verify the analyses prior to and after breaking the blinding. In addition to two-way ANOVA or unpaired t test, statistical analysis will employ stepwise multivariate regression and analysis of covariance tests in order to determine potential influence of age, gender, bypass time, drugs, body weight and other morphometric and clinical factors.

**10.4 Interim Analyses**

Due to the brief study period and small number of subjects, an interim analysis is not scheduled.

# DATA MANAGEMENT

**11.1 Data Collection**

Clinical data will be collected by Ms Hepponstall, Laboratory data will be collected by Drs Pepe and Ignjatovic and Ms Hepponstall.

**11.2 Data Storage**

Data will be stored coded on the secured laboratory computers in Heart Research and Haematology Research of MCRI.

- 1. **Study Record Retention**

Records will be maintained until the youngest participant is 25 years old.

# ADMINISTRATIVE ASPECTS

- 1. **Confidentiality**

Confidentiality is maintained per standard clinical and scientific practice at RCH/MCRI.

Samples and results are coded and stored separately to patient identifying details in a secured laboratory and office of Heamatology research, and Heart Research, Dept of Cardiology. No patient will be identified in any scientific publication or general lay report. Only patient group findings will be reported.

- 1. **Participant Reimbursement**

Not applicable.

- 1. **Financial Disclosure and Conflicts of Interest**

No financial disclosures or conflicts of interest to report.

# USE OF DATA AND PUBLICATIONS POLICY

Following conclusion of the study a lay report of the findings will be made available to all participant families, and the scientific results will be published in an international medical journal.

# TRIAL REGISTRATION

Registration of this study with the Australian Clinical Trials Registry will be completed upon Human Research Ethics Committee approval of the trial and prior to the commencement of recruitment.

# REFERENCES

1. Grund F, Gjesdal K, Kirkeboen KA, Ilebekk A. Duration of ischaemic preconditioning and importance of size of area at risk in pigs. J Mol Cell Cardiol. 1999 Jul;31(7):1369-80.

2. Jenkins DP, Pugsley WB, Yellon DM. Ischaemic preconditioning in a model of global ischaemia: infarct size limitation, but no reduction of stunning. J Mol Cell Cardiol. 1995 Aug;27(8):1623-32.

3. Teoh LK, Grant R, Hulf JA, Pugsley WB, Yellon DM. The effect of preconditioning (ischemic and pharmacological) on myocardial necrosis following coronary artery bypass graft surgery. Cardiovasc Res. 2002 Jan;53(1):175-80.

4. Kharbanda RK, Peters M, Walton B, Kattenhorn M, Mullen M, Klein N, Vallance P, Deanfield J, MacAllister R. Ischemic preconditioning prevents endothelial injury and systemic neutrophil activation during ischemia-reperfusion in humans in vivo. Circulation. 2001 Mar 27;103(12):1624-30.

5. Konstantinov IE, Arab S, Kharbanda RK, Li J, Cheung MM, Cherepanov V, Downey GP, Liu P, Cukerman E, Coles JG, Redington AN. The remote ischemic preconditioning stimulus modifies inflammatory gene expression in humans. Physiol Genomics 2004;19(1):143-50.

6. Gho BC, Schoemaker RG, van den Doel MA, Duncker DJ, Verdouw PD. Myocardial protection by brief ischemia in noncardiac tissue. Circulation. 1996 Nov 1;94(9):2193-200.

7. Birnbaum Y, Hale SL, Kloner RA. Ischemic preconditioning at a distance: reduction of myocardial infarct size by partial reduction of blood supply combined with rapid stimulation of the gastrocnemius muscle in the rabbit. Circulation. 1997 Sep 2;96:1641-6.

8. Dickson EW, Lorbar M, Porcaro WA, Fenton RA, Reinhardt CP, Gysembergh A, Przyklenk K. Rabbit heart can be "preconditioned" via transfer of coronary effluent. Am J Physiol. 1999 Dec;277(6 Pt 2):H2451-7.

9. Konstantinov IE, Li J, Cheung MM, Shimizu M, Stokoe J, Kharbanda RK, Redington AN. Prior remote ischemic preconditioning of the recipient reduces myocardial ischemia-reperfusion injury of the denervated donor heart via a Katp channel-dependent mechanism: a porcine transplant model. Transplantation 2005;79(12):1691-5.

10. Konstantinov IE, Arab S, Li J, Cheung MMH, Redington AN. The remote ischemic preconditioning modifies gene expression in mouse myocardium. J Thorac Cardiovasc Surg 2005;130(5):1326-32.

11. Kharbanda RK, Mortensen UM, White PA, Kristiansen SB, Schmidt MR, Hoschtitzky JA, Vogel M, Sorensen K, Redington AN, MacAllister R. Transient limb ischemia induces remote ischemic preconditioning in vivo. Circulation 2002 Dec 3;106(23):2881-3.

12. Schmidt MR, Smerup M, Konstantinov IE, Shimizu M, Li J, Cheung MMH, White PA, Kristiansen SB, Sorensen K, Dzavik V, Redington AN, Kharbanda RK. Intermittent peripheral tissue ischemia during coronary ischemia reduces myocardial infarction: first demonstration of remote ischemic perconditioning. Am J Physiol Heart Circ Physiol 2007; 292(4):H1883-90.

13. Kharbanda RK, Li J, Konstantinov IE, et al. Remote ischemic preconditioning protects from cardiopulmonary bypass injury in vivo. Circulation 2003;108:17(Suppl IV):IV– 509-10.

14. Kharbanda RK, Li J, Konstantinov IE, et al. Remote ischemic preconditioning protects against cardiopulmonary bypass-induced tissue injury: a preclinical study. Heart 2006;92:1506-11.

15. Shimizu M, Konstantinov IE, Kharbanda RK, et al. Effects of intermittent lower limb ischemia on coronary blood flow and coronary resistance in pigs. Acta Physiol 2007;190:103-9.

16. Cheung MM, Konstantinov IE, Kharbanda R, et al. Remote ischemic preconditioning reduces myocardial injury in children undergoing open heart surgery. Circulation 2004;110:17(Suppl III):III – 58.

17. Cheung MM, Kharbanda RK, Konstantinov IE, et al. Randomised controlled trial of the effects of remote ischemic preconditioning on children undergoing cardiac surgery: first clinical application in humans. J Am Coll Cardiol 2006;47:2277-82.

18. Hausenloy DJ, Mwamure PK, Venugopal V, et al. Effect of remote ischaemic preconditioning on myocardial injury in the patients undergoing coronary artery bypass graft surgery: a randomised controlled trial. Lancet 2007;370:575-9.

19. Saxena P, Newman MA, Redington AN, Konstantinov IE. Remote ischemic conditioning: evolution of the concept, mechanisms and clinical application. J Cardiac Surg, 2009, in press.

20. Konstantinov IE, Li J, Redington AN. From mesothelioma to myocardial protection via the PI3K pathway: a new vista in cardiothoracic surgery. J Thorac Cardiovasc Surg 2006; 131(2):509-10.

21. Konstantinov IE, Redington AN. Linking gene expression, nuclear factor kappa B, remote ischemic preconditioning and transplantation: a quest for an elusive Holy Grail or a road to an amazing discovery? J Thorac Cardiovasc Surg 2006; 131(2):507-9.

22. Wang L, Oka N, Tropak M, Callahan J, Lee J, Wilson G, et al. Remote ischemic preconditioning elaborates a transferable blood-borne effector that protects mitochondrial structure and function and preserves myocardial performance after neonatal cardioplegic arrest. J Thorac Cardiovasc Surg 2008;136:335-42.

23. Li J, Stokoe J, Konstantinov IE, Kharbanda RK, Redington AN. Evidence for a significant myocardial contribution to total metabolic burden during hypothermic cardiopulmonary bypass. Perfusion 2005;20(5):277-83.

24. Shimizu M, Saxena P, Konstantinov IE, Cherepanov V, Cheung MMH, Wearden P, et al. Remote ischemic preconditioning decreases adhesion and selectively modifies functional responses of human neutrophils. J Surg Research 2009, in press.

25. Konstantinov IE, Coles JG, Boscarino C, Takahashi M, Goncalves J, Ritter J, Van Arsdell GS. Gene expression in children undergoing cardiac surgery for right heart obstructive lesions. J Thorac Cardiovasc Surg 2004;127(3):746-54.

26. Cheung MMH, Konstantinov IE, Redington AN. Late complications of repair of tetralogy of Fallot and indications for pulmonary valve replacement. Semin Thorac Cardiovasc Surg 2005;17(2):155-9.

27. d’Udekem Y, Rubay J, Ovaert C. Failure of right ventricular recovery of Fallot patients after pulmonary valve replacement: delay of reoperation or surgical technique? J Am Coll Card 2001;37(7):2008-9.

28. d’Udekem Y, Ovaert C, Grandjean F, et al. Tetralogy of Fallot: transannular and right ventricular patching equally affect late functional status. Circulation 2000;102: III116-22.

29. d’Udekem Y, Rubay J, Shango-Lody P, et al. Late homograft valve insertion after transannular patch repair of tetralogy of Fallot. J Heart Valve Dis 1998;7(4):450-4.

30. Ali ZA, Callaghan CJ, Lim E, et al. Remote ischemic preconditioning reduces myocardial and renal injury after elective abdominal aortic aneurysm repair. A randomized controlled trial. Circulation 2007;116(Suppl I):I-98-I-105.

31. Strohn C, Barancik M, von Bruehl M, et al. Transcription inhibitor actinomycin-D abolishes the cardioprotective effect of ischemic preconditioning. Cardiovasc Res 2002; 55(3):602-18.

32. Rowland RT, Meng X, Cleveland JC, et al. Cardioadaptation induced by cyclic ischemic preconditioning is mediated by translational regulation of de novo protein synthesis. J Surg Res 1997;71(2):155-60.

33. Sheikh Am, Barrett C, Villamizar N, et al. Proteomics of cerebral injury in a neonatal model of cardiopulmonary bypass with deep hypothermic circulatory arrest. J Thorac Cardiovasc Surg 2006;132:820-8.

34. Foster DB, O’Rourke B, Van Eyk JE. What can mitochondrial proteomics tell us about cardioprotection afforded by preconditioning? Expert Rev Proteomics 2008;5(5):633-6.

35. Lull ME, Freeman WM, Myers JL, et al. Plasma proteomics: a noninvasive window on pathology and pediatric cardiac surgery. ASAIO J 2006;52(5):562-6.

36. Sheeran FL, Pepe S. Energy deficiency in the failing heart: linking increased reactive oxygen species and disruption of oxidative phosphorylation rate. Biochim Biophys Acta. 2006; 1757:543-2.

37. Rosenfeldt F, Marasco S, Lyon W, Wowk M, Sheeran F, Bailey M, Esmore D, Davis B, Pick A, Rabinov M, Smith J, Nagley P, Pepe S. Coenzyme Q10 therapy before cardiac surgery improves mitochondrial function and in vitro contractility of myocardial tissue. J Thorac Cardiovasc Surg. 2005; 129: 25-32.

38. Birch-Machin MA, Turnbull DM. Assaying mitochondrial respiratory complex activity in mitochondria isolated from human cells and tissues. Methods Cell Biol. 2001; 65: 97-117.

39. Trounce IA, Kim YL, Jun AS, Wallace DC. Assessment of mitochondrial oxidative phosphorylation in patient muscle biopsies, lymphoblasts, and transmitochondrial cell lines. Methods Enzymol. 1996; 264: 484-509.

40. Kowaltowski AJ, Cosso RG, Campos CB, Fiskum G. Effect of Bcl-2 overexpression on mitochondrial structure and function. J Biol Chem. 2002; 277: 42802-7.

41. Juhaszova M, Zorov DB, Kim SH, Pepe S, et al., Glycogen synthase kinase-3β mediates convergence of protection signaling to inhibit the mitochondrial permeability transition pore. J Clin Invest. 2004; 113: 1535-1549.

42. Sivaraman V, et al. Postconditioning protects human atrial muscle through the activation of the RISK pathway. Basic Res Cardiol. 2007;102:453-9.

43. Vermes I, Haanen C, Reutelingsperger C. Flow cytometry of apoptotic cell death. J Immunol Methods. 2000; 243:167-90.

**16. APPENDIX**

# SINGLE SERIOUS Adverse Event (SAE) REPORT FORM

- **All** Internal* SAEs (occurring to RCH participants) **must be reported to the RCH HREC within 24-72 hours of occurrence and must be accompanied by a detailed report of the event.**
- External SAEs (occurring to participants from other sites) **must be reported in a prompt manner if the information impacts the continued ethical acceptability of the trial or requires documentation to be updated (i.e. protocol or PIS).**

***An Internal SAE is one occurring in a participant that RCH researchers are responsible for, this is independent of where the event occurs.**

***Please complete this form electronically and then print.***

***Please submit 1 original & 2 copies of this form along with all supporting documentation.***

| **HREC# :** |
| --- |

| **Study Title** (inc protocol #) **:** |
| --- |

| **Date that SAE occurred :**  **Date Investigator became aware of SAE :** |
| --- |

| **Participant ID :** |
| --- |

| **Internal or External (see above definition):** |
| --- |

| **Event details (synopsis):** |
| --- |

| **Event management (synopsis):** |
| --- |

| **Event outcome (synopsis):** |
| --- |

**Investigator’s opinion of the Serious Adverse Event:**

Expected  Unexpected

**Investigator’s opinion of the Serious Adverse Event relationship to the study drug:**

**Unrelated**  **Possibly related**  **Probably Related**  **Definitely Related**

| **Other (provide details) :** |
| --- |

Does the protocol require amending as a result of this SAE? **Y**  **N**

### If Yes, please submit a modification request with the amended protocol.

Do the information/consent forms require amending as a result of this SAE? **Y**  **N**

### (If Yes, please submit a modification request with the amended forms)

**Signature Principal Investigator : ……………………………………………………………..**

Date Submitted: ………………………………………………………… ……………………….
